# Peak oxygen uptake and breathing pattern in COPD patients – a four-year longitudinal study

**DOI:** 10.1186/s12890-015-0095-y

**Published:** 2015-08-19

**Authors:** Bente Frisk, Jon A. Hardie, Birgitte Espehaug, Liv I. Strand, Rolf Moe-Nilssen, Tomas M. L. Eagan, Per S. Bakke, Einar Thorsen

**Affiliations:** Centre for Evidence-Based Practice, Bergen University College, Bergen, Norway; Department of Clinical Science, University of Bergen, Bergen, Norway; Department of Global Public Health and Primary Care, University of Bergen, Bergen, Norway; Department of Physiotherapy, Haukeland University Hospital, Bergen, Norway; Department of Thoracic Medicine, Haukeland University Hospital, Bergen, Norway; Department of Occupational Medicine, Haukeland University Hospital, Bergen, Norway

## Abstract

**Background:**

Activities of daily living in patients with chronic obstructive pulmonary disease (COPD) are limited by exertional dyspnea and reduced exercise capacity. The aims of the study were to examine longitudinal changes in peak oxygen uptake (V̇O_2peak_), peak minute ventilation (V̇_Epeak_) and breathing pattern over four years in a group of COPD patients, and to examine potential explanatory variables of change.

**Methods:**

This longitudinal study included 63 COPD patients, aged 44-75 years, with a mean forced expiratory volume in one second (FEV_1_) at baseline of 51 % of predicted (SD = 14). The patients performed two cardiopulmonary exercise tests (CPETs) on treadmill 4.5 years apart. The relationship between changes in V̇O_2peak_ and V̇_Epeak_ and possible explanatory variables, including dynamic lung volumes and inspiratory capacity (IC), were analysed by multivariate linear regression analysis. The breathing pattern in terms of the relationship between minute ventilation (V̇_E_) and tidal volume (V_T_) was described by a quadratic equation, V_T_ = a + b∙V̇_E_ + c∙V̇_E_^2^, for each test. The V_Tmax_ was calculated from the individual quadratic relationships, and was the point where the first derivative of the quadratic equation was zero. The mean changes in the curve parameters (CPET2 minus CPET1) and V_Tmax_ were analysed by bivariate and multivariate linear regression analyses with age, sex, height, changes in weight, lung function, IC and inspiratory reserve volume as possible explanatory variables.

**Results:**

Significant reductions in V̇O_2peak_ (*p* < 0.001) and V̇_Epeak_ (*p* < 0.001) were related to a decrease in resting IC and in FEV_1_. Persistent smoking contributed to the reduction in V̇O_2peak_. The breathing pattern changed towards a lower V_T_ at a given V̇_E_ and was related to the reduction in FEV_1_.

**Conclusion:**

Increasing static hyperinflation and increasing airway obstruction were related to a reduction in exercise capacity. The breathing pattern changed towards more shallow breathing, and was related to increasing airway obstruction.

## Background

Activities of daily living in patients with chronic obstructive pulmonary disease (COPD) are limited by exertional dyspnea and reduced exercise capacity [1, 2]. Exertional dyspnea appears to be related to the increased work of breathing associated with a restriction of tidal volume (V_T_) expansion [3]. A reduction in exercise capacity measured as peak oxygen uptake (V̇O_2peak_) has been found to be related to ventilatory limitations [4, 5], pulmonary gas exchange abnormalities [6], peripheral muscle dysfunction [7, 8] or any combination of these factors. COPD is a progressive disease with an increased rate of decline in forced expiratory volume in one second (FEV_1_) [9, 10]. However, the relationship between FEV_1_ and V̇O_2peak_ is considered weak, and lung function by itself is a poor predictor of exercise capacity [11].

Studies of longitudinal changes in V̇O_2peak_, peak minute ventilation (V̇_Epeak_) and breathing pattern in COPD are scarce. As far as we know, the longitudinal change in V̇O_2peak_ has only been examined in one previous study [12]. A reduction in V̇O_2peak_ over five years was found to be related to a reduction in maximal tidal volume (V_Tmax_) and V̇_Epeak_, and the decrease in V̇O_2peak_ was associated with the decrease in FEV_1_. However, only male Japanese patients were included in that study and the breathing pattern was not examined. We are not aware of any studies that have examined the longitudinal changes in V̇O_2peak_ and breathing pattern in COPD patients of Caucasian origin including both genders.

The breathing pattern in terms of the relationship between minute ventilation (V̇_E_) and V_T_ during incremental exercise has been described by Gallagher [13]. In the first phase, there is an almost linear relationship between V̇_E_ and V_T_. In the second phase, the increase in V̇_E_ is mainly caused by an increase in breathing frequency (B_f_), and a smaller increase in V_T_. In the third phase, the increase in V̇_E_ is caused by an increase in B_f_ only, and by the end of this phase, there can be a fall in V_T_. Different methods have been suggested to describe this relationship like the plateau of V_T_ and its inflection point [14], V_Tmax_ and V_T_ at a V̇_E_ of 30 L/min [15], V_T_ at given fractions of V̇_Epeak_ [16] and the Hey plot [17]. We have found that the V̇_E_-V_T_ relationship can be satisfactorily described by a quadratic model in COPD patients exercising on treadmill [18]. A quadratic model includes all available data from the exercise test. However, longitudinal changes in the breathing pattern as described by this method have not been examined.

The aims of the present study were to examine changes in exercise and ventilatory capacity and breathing pattern over four years in COPD patients, and to examine the relationship with variables that potentially contribute to explain the changes. We hypothesized that V̇O_2peak_ and V̇_Epeak_ would deteriorate during the observation period, that breathing pattern would be shallower with a lower V_Tmax_, and that the changes were related to lung hyperinflation and airway obstruction.

## Methods

### Subjects

The current study included 63 patients from the Bergen COPD Cohort Study (BCCS) who performed two cardiopulmonary exercise tests (CPETs) with an average follow-up time of 4.5 years (range 3-6 years). The BCCS was a three year follow-up study (2006–2010) and inclusion and exclusion criteria have been previously published [19]. Summarised, all patients had a smoking history of ≥ 10 pack-years, a post- bronchodilation FEV_1_ to forced vital capacity (FVC) ratio < 0.7 and a post-bronchodilator FEV_1_ < 80 % of predicted value according to Norwegian reference values [20]. All subjects in BCCS were informed of the opportunity to participate in pulmonary rehabilitation but were not otherwise actively selected or recruited for rehabilitation. There were no restrictions to treatment in the study period.

A total of 89 patients were enrolled to a 7- week pulmonary rehabilitation program, including a total of 17 sessions, during the first two years of follow-up in 2006–2008. The first CPET was performed at start of the program. The patients were invited to a second CPET in 2011/2012. At that time 26 of the 89 patients were deceased or disabled. The 63 included patients had clinically stable COPD in stages II-IV according to the Global Initiative for Chronic Obstructive Lung Disease (GOLD) (GOLD 2007) [21] and age between 44–75 years. Exclusion criteria for exercise testing were major cardiovascular disorders, a partial pressure of oxygen in arterial blood less than 8 kPa at rest, or exacerbations that required medical treatment during the last four weeks prior to testing.

### Ethics

The Western Norway Regional Research Ethics Committee approved the study. Participation in the study was voluntary. Written and oral information was given and written consent was obtained prior to inclusion.

### Spirometry

Spirometry was conducted on a Viasys Masterscope (Viasys, Hoechberg, Germany) before the CPETs according to the ATS/ERS Standardization of Lung Function Testing [22]. FEV_1_ and FVC were taken as the highest values from at least three satisfactory expiratory manoeuvers. The spirometer was calibrated before each test with a 3-L calibration syringe. The changes in FEV_1_ (ΔFEV_1_) and FVC (ΔFVC) were calculated as FEV_1_ or FVC at CPET2 minus FEV_1_ or FVC at CPET1.

### Cardiopulmonary exercise test

The patients completed two incremental CPETs to their symptom-limited maximum on a treadmill (Woodway, model: PPS 55 med Weiss, Weil am Rhein, Germany). The exercise protocol was a modified Bruce protocol [23, 24]. The test started with rest in the standing position for 2 min. The warm-up phase lasted for 1 min with a walking speed of 1.5 km h^−1^. The protocol has 20 stages, all lasting for one minute. The first stage was at 1.5 km/h with an inclination of 0 %. In stage 2, the speed was the same as in stage 1 with an inclination of 5 %. From stage 3–5, the speed increased with 0.6 km/h and the inclination was 9, 10 and 11 %, respectively. From stage 6–13, the speed increased with 0.6–0.7 km/h and the inclination with 1 %. From stage 13–14, the speed increased with 0.4 km/h, and the inclination increased with 1 %. Finally, from stage 15–20, the speed was increasing with 1 km/h each minute and the inclination was the same as in stage 14. The warm-up was not counted into the exercise time.

Blood pressure, electrocardiography (GE healthcare, Cardio Soft EKG, Freiburg, Germany) and pulse oximetry were monitored at rest, continuously during the test and for 3 min into the recovery phase. A tight-fitting oronasal mask was adjusted to each patient and checked for leaks before starting the exercise. The integrated exercise testing system (Care Fusion, V_max_ Spectra 229, Hochberg, Germany) was calibrated every morning and immediately before each test. The V_T_, B_f_, oxygen uptake (V̇O_2_), carbon dioxide output (V̇CO_2_), and heart rate (HR) were measured on a breath by breath basis and averaged over 20 s intervals. V̇_E_ and V_T_ were corrected to the body temperature pressure saturated (BTPS) condition, and V̇O_2_ and V̇CO_2_ to the standard temperature pressure dry (STPD) condition.

The patients graded their level of dyspnea and leg discomfort by Borg CR10 Scale [25]. In order to measure dynamic hyperinflation during exercise, serial measurements of inspiratory capacity (IC) were performed [5]. Measurements were taken at rest, every second minute during exercise and at peak exercise. The change in IC (ΔIC) during each of the CPETs was calculated as IC at rest minus IC at peak exercise. The change in dynamic hyperinflation between the CPETs (ΔIC_dynamic_) was calculated as ΔIC at CPET2 minus ΔIC at CPET1. The difference in IC at rest (∆IC_rest_) was calculated as IC at rest at CPET2 minus IC at rest at CPET1. The differences in V̇O_2peak_ (ΔV̇O_2peak_) and V̇_Epeak_ (ΔV̇_Epeak_) were calculated in the same way. Inspiratory reserve volume (IRV) was calculated for each of the two CPETs as the difference between IC at the end of the test minus the maximal V_T_. The difference in IRV (ΔIRV) was calculated as IRV at CPET2 minus IRV at CPET1. The estimated MVV was calculated as FEV_1_ × 35 [26].

Self-reported physical activity was recorded at baseline and after one and three years in the BCCS [27]. Two questions were related to spare time physical activity, one for strenuous and one for light physical activity. The delineation between strenuous and light was whether the activity resulted in breathlessness and sweating or not. The response categories were none, less than 1 h per week, 1-2 h per week and three or more hours per week. These questions are previously validated [28, 29]. Data on exacerbations and smoking habits were recorded at the time for the CPETs.

### Statistical analyses

Descriptive statistics were used to characterize the study population (mean, standard deviation (SD), median and percent). Independent samples t-tests for continuous variables and Pearson chi square- tests for categorical variables were used to compare patients’ characteristics and CPET responses to patients who only performed one CPET with those that completed both. Paired samples t-tests were used to analyze the longitudinal change in pulmonary function, exercise capacity and breathing pattern in the patients who completed both CPETs. Pearson correlation coefficient was used to calculate the linear relationship between the yearly change in V̇O_2peak_ and FEV_1_. The relationships between ΔV̇O_2peak_ or ΔV̇_Epeak_ and potential explanatory variables were analysed by bivariate (unadjusted) and multivariate (adjusted) linear regression analyses. Investigated variables were age, sex, height, baseline V̇O_2peak_ or baseline V̇_Epeak_, smoking during follow-up, ΔFEV_1_, ΔFVC, Δweight, ∆IC_rest,_ ΔIC_dynamic_, self-reported physical activity, exacerbations and time between the tests. ΔFVC was not included in the multivariate regression analysis, because it is an intermediary variable with ΔFEV_1_ and the outcome variable.

A quadratic model (V_T_ = a + b∙V̇_E_ + c∙V̇_E_^2^) was used to describe the relationship between V̇_E_ and V_T_ during incremental exercise_._ The goodness-of-fit for the individual patient specific regression analysis was evaluated by the adjusted coefficient of determination (adjusted R^2^) and the F statistic. For the latter a *p*-value < 0.05 was required for inclusion of the patient in further analysis. These analyses were done for each individual patient in CPET1 and CPET2.

The mean values for the curve parameters the constant (intercept) (a), the linear coefficient (slope) (b) and the quadratic coefficient (curvature) (c) were calculated. V_Tmax_ was calculated from the individual quadratic relationships, and was the point where the first derivative of the quadratic equation was zero. The mean change (CPET2 minus CPET1) in the curve parameters and the change in V_Tmax_ (ΔV_Tmax_) were analysed by bivariate and multivariate linear regression analysis with age, sex, height, Δweight, ΔFEV_1_, ΔIRV, ΔIC_rest_, and ∆IC_dynamic_ as explanatory variables.

Estimated regression coefficients are presented with 95 % confidence intervals and *p*-values. The significance level was set at 0.05. The data analyses were performed using IBM SPSS Statistics 21 (SPSS Inc. Chicago, Illinois, USA).

## Results

The baseline characteristics of the study population and the peak responses to the incremental exercise test at baseline are shown in Table 1. The patients’ mean age (SD) was 61 (6) years, 56 % were males and mean FEV_1_ in percent of predicted values was 51 (14) %. The 26 patients who only performed the first CPET were older, had lower lung function and lower peak responses at the CPET (Table 1).

**Table 1:** Baseline characteristics of the study sample (*n* = 63) compared with patients only assessed at baseline (*n* = 26)

Variables	Completed one CPET *n* = 26	Completed two CPETs *n* = 63	Group diff. *p*-value
Sex, male/female n (%)	18/8 (69/31)	35/28 (56/44)	0.232
Age (years)	64.4 ± 5.8	61.2 ± 6.2	0.028
Smoking status n (%)			0.471
Current	9 (35)	27 (43)	
Former	17 (65)	36 (57)	
Pack years	38 ± 19	43 ± 26	0.380
Height (m)	1.72 ± 0.1	1.71 ± 0.1	0.540
Weight (kg)	72.8 ± 20.7	76.4 ± 17.5	0.406
BMI (mean, kg · m^−2^)	24.4 ± 5.4	26.3 ± 4.9	0.108
FEV_1_ (% pred.)	38.2 ± 11.4	51.4 ± 13.5	<0.001
FVC (% pred.)	83.5 ± 20.6	90.1 ± 17.2	0.120
FEV_1_/FVC (%)	36.4 ± 7.6	46.4 ± 10.8	<0.001
IC_rest_ (L)	1.91 ± 0.50	2.36 ± 0.75	0.006
ΔIC(L)	0.30 ± 0.28	0.41 ± 0.40	0.232
GOLD category			0.001
II n (%)	5 (19)	34 (54)	
III n (%)	14 (54)	26 (41)	
IV n (%)	7 (27)	3 (5)	
mMRC dyspnea grade	1.6 ± 0.9	1.3 ± 1.2	0.316
Experienced^1^ ≥ 2 exacerbations last year			0.402
No n (%)	18 (69)	49 (78)	
Yes n (%)	8 (31)	14 (22)	
Exercise time (min)	4.78 ± 1.66	6.44 ± 1.88	<0.001
V̇O_2peak_ (L ∙ min^−1^)	1.09 ± 0.31	1.57 ± 0.57	<0.001
V̇CO_2peak_ (L ∙ min^−1^)	1.08 ± 0.38	1.65 ± 0.69	<0.001
V̇_Epeak_ (L ∙ min^−1^)	40.2 ± 13.3	53.8 ± 19.2	<0.001
HR_peak_ (bpm)	125 ± 21	138 ± 20	0.009
RER	0.98 ± 0.12	1.03 ± 0.12	0.047
Dyspnea (Borg Scale)	8.6 ± 1.7	9.0 ± 1.6	0.481
Leg discomfort (Borg Scale)	5.5 ± 3.4	5.6 ± 2.8	0.905
SpO_2_ start (%)	96.8 ± 3.4	97.1 ± 2.2	0.546
SpO_2_ end (%)	89.9 ± 6.3	92.8 ± 5.0	0.024

Data are presented as mean ± SD, unless otherwise stated. Chi square for categorical variables and independent *t*-test for continuous variables. CPET: cardiopulmonary exercise test; BMI: body mass index; FEV_1_: forced expiratory volume in one second; FVC: forced vital capacity; IC_rest_: resting inspiratory capacity; ΔIC: inspiratory capacity at rest minus IC at the end of the test; GOLD: Global Initiative for Chronic Obstructive Lung Disease; mMRC: modified Medical Research Council. V̇O_2peak_: peak oxygen uptake per minute; V̇CO_2peak_: peak carbon dioxide production per minute V̇_Epeak_: peak minute ventilation per minute; HR_peak_: peak heart rate; RER: respiratory exchange ratio; SpO_2:_ oxygen saturation

^1^Exacerbations requiring either hospitalization with oral antibiotics or oral steroids

Forty patients (63 %) remained in the same GOLD stage during the follow-up, while eight (13 %) patients had improved GOLD stage, six from stage III to II and two from stage IV to III, respectively. Fifteen patients (24 %) had changed to a worse GOLD stage, eight from stage II to III and seven from stage III to IV.

### Longitudinal changes in exercise and ventilatory capacity

**Fig. 1:**
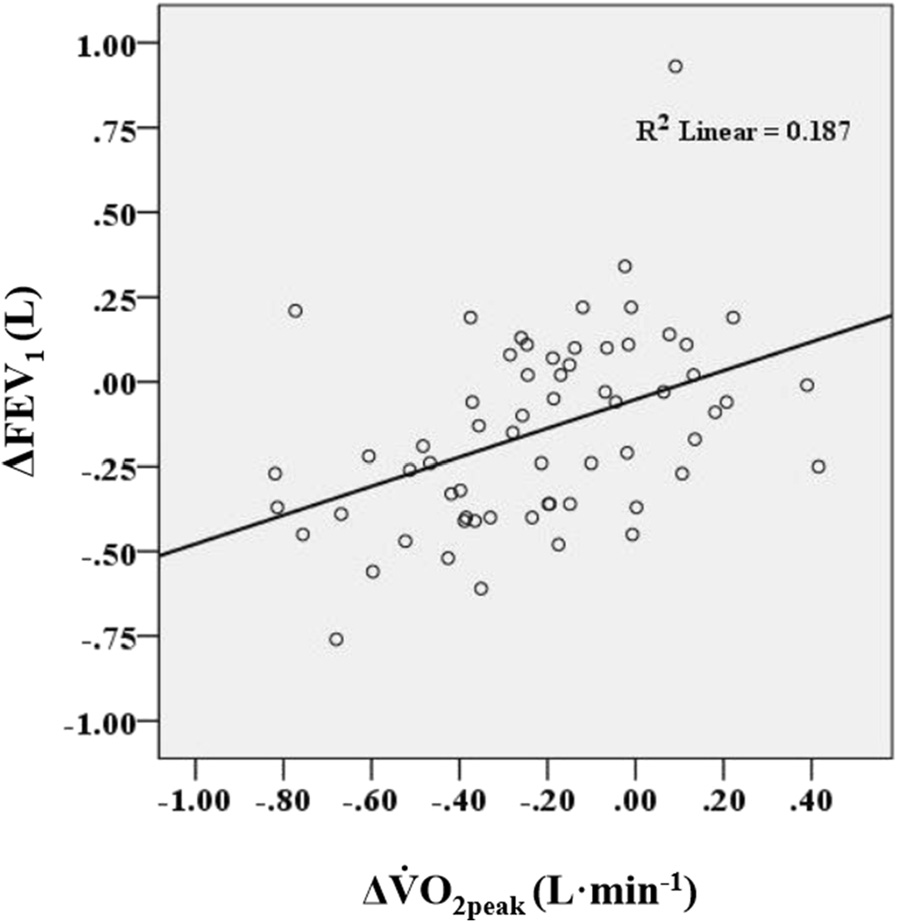
The relationship between change in V̇O_2peak_ and change in FEV_1_ between the two cardiopulmonary exercise tests (CPET). V̇O_2peak_: Peak oxygen uptake. ΔV̇O_2peak_: V̇O_2peak_ at CPET2 minus V̇O_2peak_ at CPET1. FEV_1_: Forced expiratory volume in 1 sec. Δ FEV_1_: FEV_1_ at CPET2 minus FEV_1_ at CPET1. R^2^: The coefficient of determination

FEV_1_, FVC, V̇O_2peak_ and V̇_Epeak_ decreased significantly during the follow-up period (Table 2), while the exercise time on treadmill remained constant. The Borg dyspnea score was not significantly different. The mean (SD) decline in FEV_1_ was 34 (66) mL∙yr^−1^, (*p* < 0.001) and in V̇O_2peak,_ 50 (68) mL · min^−1^ · yr^−1^ (*p* < 0.001). The decline in V̇O_2peak_ and FEV_1_ during the observation period correlated moderately (*r* = 0.43, *p* < 0.001) (Fig. 1).

**Table 2:** Pulmonary function and peak responses to cardiopulmonary exercise tests at baseline and 4.5 years apart

Variables	CPET 1 *n* = 63	CPET 2 *n* = 63	Change CPET2 minus CPET1	*p*-value
Sex, male/female (n)	35/28			
Weight (kg)	76.4 ± 17.5	76.0 ± 17.4	−0.4 ± 5.2	0.599
FEV_1_(L)	1.60 ± 0.53	1.46 ± 0.57	−0.15 ± 0.28	<0.001
FEV_1_ (% pred)	51.4 ± 13.5	48.0 ± 14.8	−3.4 ± 9.5	0.006
FVC (L)	3.47 ± 0.89	3.14 ± 0.86	−0.34 ± 0.50	<0.001
FVC (% pred)	90.1 ± 17.2	82.8 ± 15.3	−7.4 ± 14.7	<0.001
FEV_1_/FVC (%)	46.4 ± 10.8	46.0 ± 11.2	−0.4 ± 0.05	0.549
Exercise time (min)	6.44 ± 1.88	6.44 ± 2.18	−0.01 ± 1.61	0.980
V̇O_2peak_ (L ∙ min^−1^)	1.57 ± 0.57	1.36 ± 0.54	−0.22 ± 0.29	<0.001
V̇CO_2peak_ (L ∙ min^−1^)	1.65 ± 0.69	1.34 ± 0.67	−0.31 ± 0.37	<0.001
V̇_Epeak_/MVV	0.98 ± 0.22	0.94 ± 0.19	−0.03 ± 0.20	0.196
V̇_Epeak_ (L ∙ min^−1^)	53.8 ± 19.2	47.3 ± 19.6	−6.5 ± 11.6	<0.001
V_Tmax_ (L)^2^	1.71 ± 0.51	1.48 ± 0.43	−0.23 ± 0.41	<0.001
HR_peak_ (bpm)	138 ± 20	133 ± 19	−5 ± 14	0.004
RER	1.03 ± 0.12	0.96 ± 0.14	−0.08 ± 0.11	<0.001
Dyspnea (Borg Scale)	9.0 ± 1.6	8.7 ± 1.6	−0.3 ± 1.8	0.327
Leg discomfort (Borg Scale)	5.6 ± 2.8	5.9 ± 3.0	0.3 ± 2.8	0.651
IC_rest_	2.36 ± 0.75	2.21 ± 0.78	−0.13 ± 0.48	0.039
ΔIC(L)	0.41 ± 0.40	0.46 ± 0.33	0.05 ± 0.38	0.208
IRV (L)	0.44 ± 0.37	0.38 ± 0.26	−0.05 ± 0.34	0.233
MVV (L ∙ min^−1^)	56.0 ± 18.6	51.0 ± 20.1	−5.1 ± 9.8	<0.001
SpO_2_ start (%)	97.1 ± 2.2	95.9 ± 2.5	−1.2 ± 3.2	0.004
SpO_2_ end (%)	92.8 ± 5.1	89.6 ± 5.1	−3.2 ± 4.4	<0.001
Curve parameters				
Intercept (a)^1^	-0.05 (0.47)	−0.18 (0.44)	-0.13 (0.46)	0.032
Slope (b)^1^	0.063 (0.032)	0.076 (0.035)	0.014 (0.037)	0.007
Curvature (c)^1^	−0.00071 (0.00059)	−0.00105 (0.00079)	−0.00036 (0.00077)	0.002

Data are presented as mean ± SD, unless otherwise stated. Independent *t*-test for continuous variables

CPET: cardiopulmonary exercise test; FEV_1_: forced expiratory volume in one second; FVC: forced vital capacity; V̇O_2peak:_ peak oxygen uptake per minute; V̇CO_2peak_: peak carbon dioxide production per minute; V̇_Epeak_: peak minute ventilation per minute; MVV: maximal voluntary ventilation (FEV_1_x35) V_Tmax_: maximal tidal volume; HR_peak_: peak heart rate; RER: respiratory exchange ratio; IC_rest_: resting inspiratory capacity; ΔIC: IC at rest minus IC at the end of the test; IRV: inspiratory reserve volume; SpO_2:_ oxygen saturation

^1^The relationship between ventilation (V̇_E_) and tidal volume (V_T_) was described by a quadratic model (V_T_ = a + b·V̇_E_ + c·V̇_E_^2^). ^2^V_Tmax_ was calculated from the individual quadratic relationships, and was the point where the first derivative of the quadratic equation was zero

The reduction in V̇O_2peak_ was larger in subjects with a higher baseline V̇O_2peak_ (p < 0.001) and with a larger reduction in ΔIC_rest_ (*p* = 0.002) (Table 3). Furthermore age (*p* = 0.023), ΔFEV_1_ (*p* = 0.031) and smoking during follow-up (*p* = 0.021) were found to be related to the change in V̇O_2peak._ ΔV̇_Epeak_ was related to ΔIC_rest_ (*p* = 0.005), ΔFEV_1_ (*p* = 0.023) and baseline V̇_Epeak_ (*p* = 0.002) (Table 3). Gender was not associated with the reduction in V̇O_2peak_ or V̇_Epeak_.

**Table 3:** The relationships between changes in peak oxygen uptake and peak minute ventilation and explanatory variables

Variable	Unadjusted		Adjusted			*p*-value
	B	*p*-value	B	St.B*	95 % CI	
ΔV̇O_2peak_ (L ∙ min^−1^)						
Age (years)	−0.006	0.273	−0.011	−0.238	−0.020–−0.002	0.023
Sex	−0.146	0.042	0.022	0.039	−0.110–0.155	0.737
Height (m)	−0.652	0.145				
V̇O_2peak_ at baseline (L ∙ min^−1^)	−0.178	0.004	−0.260	−0.515	−0.379–−0.141	<0.001
Smoking during follow-up	−0.108	0.144	−0.139	−0.240	−0.257–−0.021	0.021
ΔFEV_1_ (L)	0.440	<0.001	0.254	0.251	0.024–0.484	0.031
ΔFVC (L)	0.271	<0.001				
Δ Weight (kg)	−0.004	0.604				
ΔIC_rest_ (L)	0.317	<0.001	0.294	0.492	0.110–0.478	0.002
ΔIC_dynamic_ (L)	0.202	0.037	−0.105	−0.140	−0.315–0.105	0.320
Strenuous physical activity	0.020	0.593				
Light physical activity	0.079	0.131				
Exacerbations	−0.100	0.166				
Time between CPET1 and CPET2	0.005	0.907				
						R^2^ = 0.567
ΔV̇_Epeak_ (L ∙ min^−1^)						
Age (years)	−0.075	0.756	−0.131	−0.070	−0.545–0.283	0.528
Sex	−2.484	0.404	3.180	0.163	−2.111–9.732	0.203
Height (m)	−3.447	0.848				
V̇_Epeak_ at baseline (L ∙ min^−1^)	−0.163	0.032	−0.259	−0.422	−0.415–−0.103	0.002
Smoking during follow-up	−3.506	0.249				
ΔFEV_1_ (L)	19.220	<0.001	11.845	0.285	1.699–21.990	0.023
ΔFVC (L)	11.626	<0.001				
Δ Weight (kg)	−0.324	0.254				
ΔIC_rest_ (L)	13.030	<0.001	12.135	0.497	3.759–20.510	0.005
ΔIC_dynamic_ (L)	11.109	0.004	−4.219	−0.137	−13.748–5.310	0.379
Strenuous physical activity	0.984	0.512				
Light physical activity	2.241	0.297				
Exacerbations	−4.775	0.104				
Time between CPET1 and CPET2	−0.299	0.870				
						R^2^ = 0.447

95 % confidence interval (CI) examined by linear regression in bivariate and multivariate analyses

CPET: cardiopulmonary exercise test; V̇O_2peak_: peak oxygen uptake per minute; ΔV̇O_2peak_: V̇O_2peak_ at CPET2 minus V̇O_2peak_ at CPET1; FEV_1_: forced expiratory volume in one second; ΔFEV_1_: FEV_1_ at CPET2 minus FEV_1_ at CPET1; FVC: forced vital capacity; ΔFVC and Δweight were calculated like ΔFEV_1._ IC: inspiratory capacity. ΔIC_rest_ was calculated as IC at rest at CPET2 minus IC at rest at CPET1. ΔIC was calculated as IC at rest minus IC at the end of the test for both CPET1 and CPET2. ΔIC_dynamic_ was calculated as ΔIC at CPET2 minus ΔIC at CPET1; V̇_E_: minute ventilation per minute; ΔV̇E_peak:_ peak V̇_E_ at CPET2 minus peak V̇_E_ at CPET1; R^2^: The coefficient of determination

*St.B: Standardised beta

Self-reported physical activity during follow-up as reported in Bergen COPD Cohort Study and exacerbations were not related to the longitudinal changes in V̇O_2peak_ or V̇_Epeak_ (Table 3).

### Longitudinal changes in breathing pattern

**Fig. 2:**
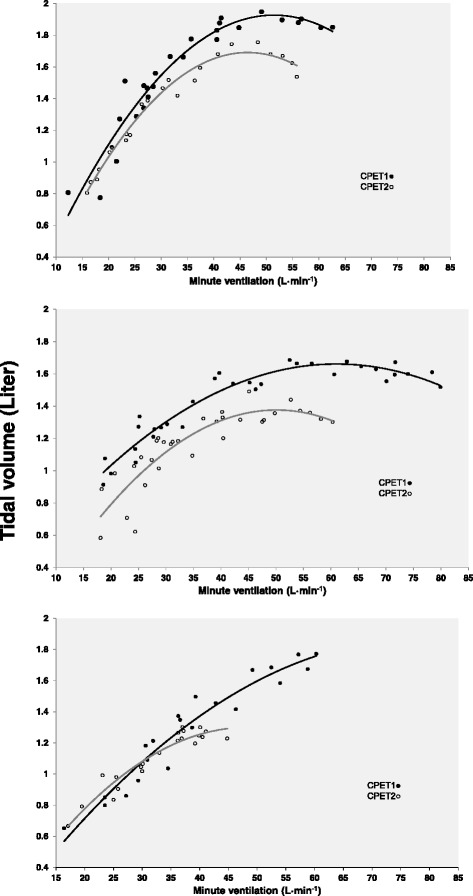
A random set of three individual responses from the two CPETs performed 4.5 years apart. CPET: Cardiopulmonary exercise test

The quadratic model described the relationship between V̇_E_ and V_T_ in 61 of 63 patients at CPET1 and at 59 of 63 patients in CPET2. In these subjects the p-value of the F-statistics for the quadratic model was <0.05 and the R^2^ ranged from 0.35 to 0.99 (median 0.90) at CPET1, and from 0.40 to 0.98 (median 0.90) at CPET2. A random set of three individual responses from CPET1 and CPET2 are shown in Fig. 2. For the six excluded patients the goodness of fit was not statistically significant and the exercise time was short with few observations. The means of the estimated constant (a), the linear coefficient (b) and the quadratic coefficient (c) changed significantly from CPET1 to CPET2 (Table 2). The linear coefficient (b) increased (*p* = 0.007) and the quadratic coefficient (c) decreased (*p* = 0.002). The changes in the curve parameters were all related to ΔFEV_1_, but not to the other possibly explanatory variables (Table 4). Maximal V_T_ was achieved at a lower V̇_E_.

**Table 4:** The relationships between the change in the curve parameters^1^ and explanatory variables (CPET2 minus CPET1)

	Unadjusted			Adjusted		
Variable	B	*p*-value	B	St.B*	95 % CI	*p*-value
Intercept^1^ (Curve parameter *a*)						
Age	0.002	0.803	−0.0003	−0.004	−0.015–0.014	0.966
Sex	−0.089	0.468	−0.126	−0.138	−0.312–0.060	0.180
Height	0.145	0.844				
a_baseline_	−0.545	<0.001	−0.573	−0.588	−0.769–−0.376	<0.001
Δweight	−0.002	0.881				
ΔFEV_1_	0.601	0.005	0.762	0.461	0.376–1.149	<0.001
ΔFVC	0.427	0.001				
ΔIC_rest_ (L)	0.116	0.374	−0.052	−0.053	−0.365–0.262	0.743
ΔIC_dynamic_ (L)	−0.025	0.882	−0.203	−0.166	−0.561–0.156	0.262
ΔIRV	−0.147	0.416				
						R^2^ = 0.507
Slope^1^ (Curve parameter *b*)						
Age	−0.0003	0.725	0.00003	0.006	−0.001–0.001	0.959
Sex	0.002	0.860	0.012	0.159	−0.005–0.029	0.177
Height	−0.059	0.311				
b_baseline_	−0.568	<0.001	−0.588	−0.510	−0.854–−0.322	<0.001
Δweight	0.002	0.862				
ΔFEV_1_	−0.046	0.009	−0.050	−0.373	−0.085–−0.015	0.006
ΔFVC	−0.032	0.003				
ΔIC_rest_ (L)	−0.008	0.429	−0.001	−0.008	−0.029–0.028	0.963
ΔIC_dynamic_ (L)	0.005	0.734	0.018	0.183	−0.014–0.050	0.265
ΔIRV	−0.023	0.119				R^2^ = 0.393
Curvature^1^ (Curve parameter *c*)						
Age	−4.8x10^−6^	0.774	−7.2x10^−6^	−0.061	−0.00004–0.00002	0.643
Sex	−7.3x10^−5^	0.746	−2.5x10^−5^	−0.016	−0.0004–0.0004	0.901
Height	0.001	0.586				
c_baseline_	−0.406	0.033	−0.345	−0.254	−0.695–0.006	0.054
Δweight	7.5x10^−6^	0.762	B			
ΔFEV_1_	0.001	0.001	0.001	0.372	0.0002–0.002	0.013
ΔFVC	0.001	<0.001				
ΔIC_rest_ (L)	0.0005	0.025	0.0004	0.268	−0.00002–0.001	0.192
ΔIC_dynamic_ (L)	0.0001	0.651	−0.001	−0.249	−0.001–0.0002	0.179
ΔIRV	−0.0003	0.280				
						R^2^ = 0.315

95 % confidence interval (CI) examined by linear regression in bivariate and multivariate analyses

CPET: cardio pulmonary exercise test; Δweight: weight at CPET2 minus weight at CPET1; FEV_1_: forced expired volume in one second, ΔFEV_1_: FEV_1_ at CPET2 minus FEV_1_ at CPET1; FVC: forced vital capacity; ΔFVC: FVC at CPET2 minus FVC at CPET1; IC: inspiratory capacity; ΔIC_rest_ was calculated as IC at rest at CPET2 minus IC at rest at CPET 1; ΔIC was calculated as IC at start of the test minus IC at the end of the test; ΔIC_dynamic_ was calculated as ΔIC at CPET2 minus ΔIC at CPET1; IRV: inspiratory reserve volume; ΔIRV was calculated as IRV at CPET2 minus IRV at CPET1; a_baseline:_ curve parameter a at baseline; b_baseline_: curve parameter b at baseline; c_baseline_: curve parameter c at baseline; R^2^: The coefficient of determination

^1^ The relationship between ventilation (V̇_E_) and tidal volume (V_T_) was described by a quadratic model (V_T_ = a + b·V̇_E_ + c·V̇_E_^2^), and the a, b, and c were calculated as the difference between CPET2 minus CPET1

*St.B: Standardised beta

The V_Tmax_ decreased significantly from CPET1 to CPET2 (*p* < 0.001) (Table 2). In the multivariate linear regression analysis, ΔV_Tmax_ was significantly related to the reduction in FEV_1_ (B = 0.429, CI: 0.106–0.751, *p* < 0.010), the reduction in IC_rest_ (B = 0.325, CI: 0.053–0.596, *p* < 0.020) and the baseline V_Tmax_ (B = –0.471, CI: –0.662––0.281, *p* < 0.01).

## Discussion

The major findings of this study of a group of COPD patients who were followed over a mean time of 4.5 years were a reduction in V̇O_2peak_ and V̇_Epeak_ which were related to a decrease in resting IC and FEV_1_, and persistent smoking during the observation period. The breathing pattern changed towards a lower V_Tmax_ and a lower V_T_ at a given V̇_E_. The reduction in FEV_1_ was related to the changes in the curve parameters describing the breathing pattern.

The mean reduction in V̇O_2peak_ was 50 (SD = 68) mL∙min^−1^∙yr^−1^, which was slightly higher than the decline in V̇O_2peak_ of 32 mL∙min^−1^∙yr^−1^ in male COPD patients reported by Oga et al. [12]. In our study, both genders were included, but the reduction in V̇O_2peak_ or V̇_Epeak_ was not associated with gender. The reduction in resting IC and FEV_1_, baseline V̇O_2peak_, smoking during follow-up and age were all found to be related to the change in V̇O_2peak_. As shown in Fig. 1 the association between the changes in FEV_1_ and V̇O_2peak_ was rather weak with large interindividual variation. The strongest associations were found for resting IC and baseline V̇O_2peak_. Total lung capacity (TLC) was not measured, and we are not aware of any studies having described the longitudinal change in TLC in COPD patients. Based on data from cross-sectional studies of COPD patients, TLC is expected to remain unaltered or slightly increased [30–32]. Thus, there is a possibility that the increase in static hyperinflation as estimated by resting IC is underestimated. There were no significant differences in dynamic hyperinflation between CPET1 and CPET2. Without knowledge about TLC changes in dynamic hyperinflation may have been obscured. Theoretically, dynamic hyperinflation is likely important since it is associated with increased work of breathing and dyspnea [1, 33].

The exercise time was the same at the two CPETs, which can indicate better working economy, even though V̇O_2peak_ and lung function decreased significantly. The respiratory exchange ratio and V̇CO_2peak_ were lower at the second CPET, whereas maximal Borg dyspnea score was not significantly different at the two CPETs. These observations are consistent with previous studies showing that laboratory-based constant work rate tests can be more sensitive demonstrating improvements after interventions than V̇O_2peak_ [11, 34].

We described the breathing pattern in the individual patient by a quadratic relationship between V̇_E_ and V_T_. The relationship could be satisfactorily described by the model in the majority of the subjects, and the model accounts for all observations throughout the exercise test. The limitations with other methods [14–17] are that all observed data from the exercise test are not included in the analyses. O’Donnell et al. [14] has described the relationship between V̇_E_ and V_T_ during exercise as linear until an inflection point. After this point, further increase in V̇_E_ is accomplished by an increase in B_f_. The inflection point is determined “by eye” by two or three persons independently of each other [14, 17]. When we examined our exercise data, it was not obvious to see where an inflection point could occur. In a quadratic model, which is analysed mathematically, the curve parameter c in the equation will be related to a “perceived” inflection point as it describes the “sharpness” of the curvature, and the V_Tmax_ can be calculated by derivation of the equation. By using this method determinations of the curve parameters will not be influenced by intra-and/or inter observer reliability. Another consideration is that during an incremental exercise test, there will be a gradual transition between the phases as described by Gallagher [13], and determining a cut-off point where the change from one phase to the other takes place, is not exactly defined. The changes in the curve parameters describing the breathing pattern were found to be related to the change in FEV_1_.

The time constant for the lung is increased in COPD due to both increased resistance and compliance. Dynamic hyperinflation in response to increasing ventilatory demands is a necessary compensatory mechanism allowing complete respiratory cycles. Resistance and compliance are both related to FEV_1_, but we did not find any relationships between changes in breathing pattern and changes in IC or IRV after adjusting for FEV_1_.

### Study limitations

The BCCS, which this study sample is a part of, made no restrictions to treatment in the study period, and the participants were free to receive medication or other treatment prescribed by their physician. Since the included patients participated in a pulmonary rehabilitation program after the first CPET, this was a selected group and the results cannot be generalized to the common COPD population. One of the major effect of pulmonary rehabilitation is to increase exercise tolerance and reduce shortness of breath, but since there was no maintenance program, the effect of the rehabilitation probably would be negligible after four years. The reduction in FEV_1_ in our patients was 34 (SD = 66) mL · year^−1^, which was not different from the mean rate of decline in FEV_1_ of 33 mL · year^−1^ in the ECLIPSE study [10]. Without participation in pulmonary rehabilitation, the decline in V̇O_2peak_ and V̇_Epeak_ could have been larger, resulting in an even stronger association with the predictors.

Self-reported physical activity was not measured synchronized with the second CPET, but was part of the data collection at baseline, and at one and three year follow-up in BCCS. We have previously shown that patients who participated in pulmonary rehabilitation were more physically active than those who had not [27]. We assume that the physical activity level would not be substantially different from this recording.

The dropout rate from baseline to follow-up after a mean time of 4.5 years was 29 %. The patients were lost to follow-up mainly because of death or disease severity. In the study of Oga et al. [12] the dropout rate was 51 %. In longitudinal observational studies with the 6 min walking distance as the main outcome and a follow-up period of 3–5 years, the dropout rate varied between 31–34 % [35–37]. With a progressive disease, an increasing dropout rate over time is unavoidable and as compared with previous studies our dropout rate is not considered high.

## Conclusions

There was a significant reduction in V̇O_2peak_ and V̇_Epeak_ over 4.5 years in this group of COPD patients. The changes were related to an increase in lung hyperinflation and a reduction in FEV_1_ along with persistent smoking during the study period. The breathing pattern changed towards a lower V_Tmax_ and a lower V_T_ at a given V̇_E_ and the reduction in FEV_1_ predicted these changes. The findings indicate that optimal treatment of airway obstruction and lung hyperinflation, and smoking cessation are all important in optimizing functional capacity in COPD along with physical training programs.
